# Steroid Resistance/Dependence Might Be an Alarming Feature for Cytomegalovirus Infection Among Ulcerative Colitis Patients With Increased Disease Activity

**DOI:** 10.7759/cureus.30873

**Published:** 2022-10-30

**Authors:** Emre Gerçeker, Fatih Saygılı, Arzu Avcı, Hakan Yuceyar

**Affiliations:** 1 Gastroenterology, İzmir Medicana International Hospital, İzmir, TUR; 2 Pathalogy, Katip Çelebi University Atatürk Training and Research Hospital, İzmir, TUR

**Keywords:** ulcerative colitis, steroid resistance, steroid dependence, flare-up, cytomegalovirus

## Abstract

Background/Aims

This study aimed to determine the prevalence of cytomegalovirus (CMV) infection among patients with moderate to severe active ulcerative colitis (UC) and to determine the risk factors for CMV infection according to the demographic features of these patients.

Patients/Methods

A total of 183 patients with severe or moderate active UC were enrolled in the study after retrospective analysis. The disease severity of UC was determined according to the Mayo Score. CMV infection was investigated by real-time quantitative polymerase chain reaction (PCR) and the immunohistochemical (IHC) staining method in colonic mucosal biopsies.

Results

CMV infection was diagnosed in 33.9% of patients with UC. UC patients diagnosed with CMV infection had significantly higher Mayo Score levels (9.68 vs 8.56 and p=0.001). The long-term presence of UC disease, steroid, azathioprine (AZA), and anti-tumor necrosis factor-alpha (anti-TNF-alpha) usage increased the risk of CMV infection (p=0.001 and odds ratio=1.168; p=0.001 and odds ratio=2.967; p=0.004 and odds ratio=2.953; p=0.003 and odds ratio=3.861, respectively). CMV infection increases the risk of developing steroid resistance or dependency (p=0.002 and odds ratio=3.147; p=0.002 and odds ratio=5.085, respectively). Post-treatment clinical remission and mucosal healing rates were higher in CMV-negative patients than in CMV-positive patients (99.2% vs 91.9%, p=0.018 and 86.8% vs 70.9%, p=0.015). A higher rate of need for colectomy had been found in patients with CMV infection (5 patients vs 1 patient; p=0.034 and odds ratio=10.526).

Conclusions

The presence of CMV infection increases the severity of the disease and worsens clinical outcomes, leading to adverse treatment outcomes. CMV infection increases the requirement for colectomy. The presence of steroids, immunosuppressives such as AZA, and anti-TNF-alpha usage increases the occurrence of CMV infection. CMV infection should be suspected in patients with moderate to severe UC activity.

## Introduction

Ulcerative colitis (UC) is a chronic inflammatory disease of the colon characterized by relapses and remissions. In UC therapy, the main goal is to ensure and maintain clinical remission and mucosal healing. The most important target of clinicians is also to improve the quality of life of patients and maintain safety. Prevention or reduction of disease complications, reduction of drug toxicity, a requirement for hospitalization, and elimination of the need for surgical intervention are also intended [1,2]. UC exacerbations impair a person's quality of life and lead to significant morbidity, including colectomy and mortality. Although the pathogenesis of disease exacerbation is not fully understood, many causes lead to UC exacerbation. It is known that the use of antibiotics or nonsteroidal anti-inflammatory drugs in bacterial or viral infections, such as cytomegalovirus (CMV), is associated with the exacerbation of UC [3].

The first reports that showed the relationship between CMV and UC were published in 1961 [4]. However, the pathological and clinical consequences of CMV infection remained controversial in IBD patients for many years. The role of CMV infection in patients with UC is not fully proven yet. It is concluded that CMV is a frequent cause of refractory colitis [5]. CMV is a member of Herpesviridae. First, lesions due to a CMV infection may be due to primary infection as well as reactivation of a latent virus. Second, these lesions may develop when a seropositive patient is reinfected via transplantation or blood transfusion [6]. We know that primary infections tend to be asymptomatic in patients with normal immunity. Early childhood is the most common time for CMV exposure. These persons are frequently asymptomatic [6]. After initial infection, CMV resides latently in monocytes, fibroblasts, myeloid cells, and endothelial cells. If reactivation may occur, CMV lesions may develop by activated proinflammatory cytokines such as tumor necrosis factor-alpha (TNF-alpha) and catecholamine [6]. CMV reactivation in immunocompromised patients can cause severe complications. Retinitis, pneumonia, and colitis may appear. The gastrointestinal lesions of CMV infection can be observed anywhere from the mouth to the rectum. However, colonic involvement in a CMV infection is the most common site [7-10]. CMV infection is widely spread in all parts of the world. It is more common in Asia, South America, and Africa than in western countries. The seroprevalence of CMV ranges from 45% to 100% [11]. In Turkey, CMV seroprevalence has been investigated in various groups and seropositivity has been reported between 85% and 100% [12].

The primary aim of this study is the detection of the CMV infection rate in severe and moderately active ulcerative colitis by a mucosal polymerase chain reaction (PCR) and the immunohistochemical (IHC) staining method. The second aim of the study is to determine the risk factors leading to the development of CMV infection according to the demographic characteristics findings of patients with UC.

## Materials and methods

Study design and patients

The study was carried out in the gastroenterology department of Izmir Medicana International Hospital. Before starting the study, approval was obtained from the Ethics Committee of Karatay University (IRB approval number: 2022/043). One-hundred eighty-three (n=183) patients over the age of 18 who were admitted to our clinic with a diagnosis of severely or moderately active ulcerative colitis according to the Mayo Score between 2020 and 2022, and who were tested with CMV PCR and IHC staining for the diagnosis of CMV infection, were included in the study. The diagnosis of UC was established by using clinical, endoscopic, histologic, and radiologic parameters. In all patients, age, gender, diagnosis of other concomitant systemic diseases, the extent of disease involvement, duration of disease, disease activation severity, extra-intestinal involvement, and medical treatment for the disease were retrospectively scanned and recorded. Complete blood count (CBC) and routine biochemical and imaging tests, such as esophagogastroduodenoscopy, colonoscopy, abdominal computed tomography, and magnetic resonance imaging performed at the time of admission were retrospectively scanned and recorded. The severity of the disease was determined according to the Mayo clinical scoring system. A Mayo Score between 6 and 10 was considered moderate and a Mayo Score of 11-12 was considered a severe disease. Serum antigen, serum antibody, serum or stool culture, blood and tissue PCR tests for infectious diseases that may appear simultaneously with UC, such as CMV, tuberculosis, entamoeba histolytic, clostridium difficile performed in the microbiology and biochemistry laboratory of Medicana International Hospital. All test results were retrospectively scanned and recorded. The cases were excluded from the study if they were positive for tuberculosis, entamoeba histolytica, and clostridium difficile.

Clinical follow-up and medical treatment

After the initial evaluation, medical treatment was initiated for all patients. Medical treatment in CMV-negative patients was arranged according to European Crohn´s and Colitis Organisation (ECCO) guidelines. All CMV-positive patients were treated with 5-7.5 mg/kg/day ganciclovir intravenously for 21 days. Once detection of CMV positivity was observed, azathioprine (AZA) and anti-TNF-alpha treatments were stopped. Severe cases were treated and followed up in the hospital setting. Moderate cases were followed up in an outpatient clinic once a week for the first month and every four weeks thereafter. All patients were re-evaluated clinically, in the laboratory, and endoscopically during the follow-up period. Cases that did not have abdominal pain, rectal bleeding, or diarrhea and had decreased CRP levels in laboratory tests were accepted as in clinical remission. After the start of the treatment, mucosal healing was evaluated by colonoscopy at the earliest six weeks, at the latest 12 weeks. Cases with a Mayo Score ≤ 2 were considered mucosal healing.

Steroid Resistance/Dependence

Steroid resistance was defined as active disease despite prednisone up to 0.75-1.00 mg/kg per day over a period of at least four weeks. Steroid-dependent patients were defined as those who are either unable to reduce steroids below the equivalent of prednisolone 10 mg/day within three months of initiation without recurrent active disease or who have a relapse within three months after the end of the therapy [1,2].

CMV Infection

IHC staining, hematoxylin-eosin (H&E) staining, and CMV DNA PCR analysis of colonic tissue biopsy samples were used in the diagnosis of CMV. If a patient with UC had a high tissue CMV PCR level (>250 copies/mg of tissue) and high IHC staining (>four cells/ section), the patient was considered to have CMV infection. Mucosal healing was checked in colonoscopy examination after antiviral treatment. A simultaneous mucosal biopsy was taken to detect CMV clearance in colon tissue. CMV DNA PCR determination and immunohistochemical staining were performed in the tissue. If PCR and IHC staining were negative, the cure was accepted.

Statistics

Parameters were made by using the SPSS 22 for Windows statistics program (IBM Corp., Armonk, NY). Categorical (nominal) values were expressed as percentages (%) and compared with the chi-square test [2]. Continuous numerical (quantitative) values were expressed as mean ± standard deviation (SD). Quantitative variables were compared with the student's t-test. To identify candidate risk factors for CMV infections with UC, univariate analyses were conducted using Fisher’s exact test. All factors that were significant on univariate analysis were entered into multivariate logistic regression models constructed to identify significant independent risk factors for CMV infection. The results are expressed as odds ratios (ORs) with 95%CIs. p <0.05 was determined as statistically significant.

## Results

Basic clinical and demographic data of all patients are summarized in Table 1. The mean age of 183 patients with severe and moderately active UC was found to be 41.56 ± 12.90 (18-79). Fifty-seven point nine percent (57.9%) of the patients were male. The mean duration of illness was 5.71 ± 4.61 (0-18) years. Forty-six point four percent (46.4%; n = 85) of the patients had left colon involvement, and 53.6% (n = 98) had pancolitis. Sixty-six point one percent (66.1%; n = 121) patients had moderate UC and 33.9% (n = 62) of them had severe activity. The mean Mayo Score was 8.94 ± 1.58 (5-12). Seventy-six percent (76.0%) of patients (n=139) were using oral mesalazine. Forty-five point four (45.4%) of patients (n=83) had used steroids before. Steroid resistance was observed in 21.9% (n=40) of the patients who received steroid therapy for remission induction. Fifty-seven point five percent (57.5%) of these patients (n=23) had a previous history of steroid use due to UC exacerbation. Ten point four percent (10.4%; n=19) of the patients had steroid dependence. Fifty-seven point nine percent (57.9%) of these patients (n=11) had a previous history of steroid use due to UC exacerbation. Twenty-two point four percent (22.4%; n=41) of the patients were using AZA and 14.2% (n=26) were using anti-TNF-alpha agents. Two point two percent (2.2%; n=4) of patients with UC were using AZA and anti-TNF-alpha agents together. The patients treated with AZA and anti-TNF-alpha did not use steroids. CMV infection was detected in 62 (33.9%) of 183 patients. The CMV infection rate was 7.9% (n=3) in patients with newly diagnosed ulcerative colitis. In our study, among patients with steroid-sensitive UC, the prevalence of CMV infection was 21.8%. Among patients diagnosed with steroid-resistant UC was 55%. Among patients with steroid-dependent UC, the prevalence of CMV infection was 68.4%. Among patients using AZA, the prevalence of CMV infection was 53.7%. Among patients using anti-TNF-alpha, the prevalence of CMV infection was 61.5%. Viral load assessed in the colonic mucosa of UC patients ranged from 94754.42 ± 196538.87 (700-1211000) genome copies/ml total extracted DNA.

**Table 1:** Demographic and clinical features of patients with UC (n=183) Demographic and clinical features of patients with UC (n=183) were presented. Anti-TNF-alpha: anti-tumor necrosis alpha, AZA: azathioprine, CMV: Cytomegalovirus, IHC: immunohistochemical staining, PCR: polymerase chain reaction

Gender	Male	57.9% (n=106)
	Female	42.1% (n=77)
Age		41.56 ± 12,90 (18-79)
Duration of disease (year)		5.71 ± 4.61 (0-18)
Disease extension	Left colitis	46.4% (n=85)
	Pancolitis	53.6% (n=98)
Disease severity	Moderate	66.1% (n=121)
	Severe	33.9% (n=62)
Mayo score		8.94 ± 1.58 (5-12)
Treatment	Mesalazine	76.0% (n=139)
	Steroid	45.4% (n=83)
	Steroid resistance	21.9% (n=40)
	Steroid dependence	10.4% (n=19)
	AZA	22.4% (n=41)
	Anti-TNF-alpha	14.2% (n=26)
	AZA + Anti-TNF-alpha	2.2% (n=4)
CMV IgM positive		0% (n=0)
CMV IgG positive		67.2% (n=123)
Inclusion body positive		8.7% (n=16)
CMV IHC positive		33.9% (n=62)
CMV PCR (+)		33.9% (n=62)
Viral load (genome copies/ml)		94754.42 ± 196538.87 (700-1211000)

The main clinical and demographic data of CMV infection negative and positive patients are summarized in Table 2. There was no significant difference in terms of age and gender between the CMV positive and negative groups. UC patients diagnosed with CMV infection had significantly longer disease duration (p=0.001). More cases of pancolitis were observed in the CMV-positive group. (p=0.006). UC patients diagnosed with CMV infection had significantly higher CRP level, WBC level, and Mayo Score than those without CMV infection (41.19 mg/dl vs 31.35 mg/dl and p=0.003; 10.900 vs 9.117 and p=0.001; 9.68 vs 8.56 and p=0.001, respectively). Steroid usage, steroid resistance, steroid dependence, azathioprine, and anti-TNF-alpha usage were significantly higher in UC patients with CMV infection than in UC patients without CMV infection.

**Table 2:** Demographic and clinical features of CMV (+) and CMV (-) patients with UC Demographic and clinical features of CMV (+) and CMV (-) patients with UC were presented. CMV (+) and CMV (-) groups were compared. Anti-TNF-alpha: antitumor necrosis alpha, AZA: azathioprine, CMV: Cytomegalovirus, CRP: C-reactive protein

		CMV (-)	CMV (+)	P value
Gender	Male	55.3% (n=67)	62.9% (n=39)	0.329
	Female	44.7% (n=54)	37.1% (n=23)	
Age		41.01 ± 12.59	42.65 ± 13.52	0.418
Duration of disease (year)		4.67 ± 4.30	7.74 ± 4.53	0.001
Disease extension	Left colitis	53.7% (n=65)	32.3% (n=20)	0.006
	Pancolitis	46.3% (n=56)	67.7% (n=42)	
Disease severity	Moderate	78.5% (n=95)	41.9% (n=26)	0.001
	Severe	21.5% (n=26)	58.1% (n=36)	
Mayo Score		8.56 ± 1.49	9.68 ± 1.49	0.001
CRP (mg/dl)		31.35 ± 21.10	42.19 ± 27.32	0.003
Leukocyte		9.117 ± 2.517	10.900 ± 4.297	0.001
Treatment	Steroid	36.4% (n=44)	61.9% (n=39)	0.001
	Steroid resistance	14.8% (n=18)	35.4% (n=22)	0.002
	Steroid dependence	4.9% (n=6)	20.9% (n=13)	0.002
	AZA	15.7% (n=19)	35.5% (n=22)	0.004
	Anti-TNF-alpha	8.2% (n=10)	25.8% (n=16)	0.003
CMV IgM positive		0% (n=0)	0% (n=0)	
CMV IgG positive		47.1% (n=57)	100% (n=62)	
Clinical remission		99.2% (n=120)	91.9% (n=57)	0.018
Mucosal healing		86.8% (n=105)	70.9% (n=44)	0.015
Colectomy		0.8% (n=1)	8.1% (n=5)	0.018

When evaluated with binary logistic regression analysis (Table 3), it was determined that the presence of CMV infection increased the severity of the disease and caused UC with wider area involvement (p=0.001 and odds ratio=5.052; p=0.006 and odds ratio=2.437, respectively). It was also observed that the presence of CMV infection increased steroid resistance and steroid dependence (p=0.002 and odds ratio=3.147; p=0.002 and odds ratio=5.085, respectively).

**Table 3:** Effects of CMV infection on ulcerative colitis Effects of CMV infection on ulcerative colitis activity level and treatment outcome were analyzed with logistic regression analysis.

		%95 Confidence interval		
	Odds Ratio	Lower	Upper	P value
Disease Severity	5.052	2.601	9.841	0.001
Disease Extend	2.437	1.284	4.629	0.006
Treatment				
Steroid resistant (+)	3.147	1.529	6.479	0.002
Steroid depended (+)	5.085	1.827	14.151	0.002
Colectomy	10.526	1.202	92.198	0.034
Clinical remission	0.095	0.011	0.832	0.034
Mucosal healing	0.372	0.174	0.796	0.011

Post-treatment clinical remission and mucosal healing rates were higher in CMV-negative patients than in CMV-positive patients (p=0.018 and p=0.015) (Table 2). Clinical remission was observed during clinical follow-up in 99.2% of 121 patients with CMV-negative UC. The mucosal healing rate was 86.8% (n=105). Remission was not observed in one patient (0.8%) and colectomy was required. All CMV-positive patients were treated with 5-7.5 mg/kg/day ganciclovir intravenously for 21 days. No patients experienced significant side effects from the antiviral therapy. Remission with antiviral ganciclovir therapy was observed in 57 (91.9%) of 62 patients with CMV-positive UC. The mucosal healing rate was 70.9% (n=44). Remission was not observed in five patients (8.1%) despite ganciclovir treatment, and these patients required colectomy. As a result of binary logistic regression analysis, it was observed that the presence of CMV infection decreased the rates of clinical remission and mucosal healing (p=0.034 and odds ratio=0.095; p=0.011 and odds ratio=0.372, respectively). UC patients with CMV infection had more colectomy prevalence than patients without CMV (8.1% vs 0.8%; p=0.034 and odd ratio=10.526) (Table 3).

Table 4 summarizes the risk factors for the development of CMV infection. At the end of the evaluation with multivariate logistic regression analysis, it was observed that age and gender did not increase the risk of developing CMV infection (p=0.742 and odds ratio=0.996; p=0.329 and odds ratio=1.367, respectively). The long-term presence of UC disease, steroid, AZA, and anti-TNF-alpha usage increased the risk of CMV infection (p=0.001 and odds ratio=1.168; p=0.001 and odds ratio=2.967; p=0.004 and odds ratio=2.953; p=0.003 and odds ratio=3.861, respectively).

**Table 4:** Risk factors of CMV infection Risk factors for CMV infection were analyzed with multivariate logistic regression analysis. TNF: tumor necrosis factor, AZA: azathioprine, CMV: Cytomegalovirus

		CMV(+/-)		
		%95 Confidence interval		
	Odds Ratio	Lower	Upper	P value
Age	0.996	0.970	1.022	0.742
Gender	1.367	0.730	2.560	0.329
Disease duration	1.168	1.083	1.259	0.001
Steroid	2.967	1.537	5.597	0.001
AZA	2.953	1.445	6.033	0.004
Anti-TNF-alpha	3.861	1.631	9.138	0.003

## Discussion

The relationship between CMV infection and UC has been known for a long time. Dormant CMV stays silent in the latent phase inside myeloid progenitor and endothelial cells. After a decrease in the host immune defense due to many different factors, CMV can be reactivated [6]. The prevalence of CMV infection is determined to be very high in steroid-resistant UC. It is not definitely known whether CMV is a cause of reactivation or a consequence of relapse or therapeutic refractoriness to immunosuppressants (corticosteroids and AZA) or immunomodulators (anti-TNF-alpha) [6-12]. The activation of CMV can worsen the clinical outcomes of UC [13]. In our study, similar to the data of previous studies, it was observed that CMV-positive cases had a higher Mayo Score, and the frequency of pancolitis was higher. In addition, it was observed that CMV-positive cases had higher rates of resistance to treatment, less clinical remission, and mucosal healing in line with the literature. We think that evaluation for CMV is necessary for patients with severe UC activity because it changes the course of the disease.

In two studies, the prevalence of CMV antibodies was observed in 37-97% of the adult population [14,15]. In a Turkish population, authors offered that CMV seroepidemiology in Turkey differs as socioeconomic changes occur. The prevalence is very high (97%) and the changes in CMV serostatus are different [16]. In our study, the prevalence of CMV infection in patients with severe and moderately active UC was found to be 33.9%. Studies including severely active UC patients reported that the prevalence of CMV infection ranged from 16% to 34% according to various diagnostic serological and histological methods [17-18]. There is a higher CMV seropositivity in our country than in the world. Similar to the high level of CMV seropositivity in our country, colonic CMV PCR positivity close to the upper limit in the literature was observed in our study. Moreover, although there were both moderately and severely active UC cases in our study. CMV infection rate was found to be low, similar to the studies in the literature.

In our study, according to multivariate analysis, it was observed that the long-term presence of UC disease, steroids, AZA, and anti-TNF-alpha usage increased the risk of CMV infection. However, age and gender did not increase the risk of developing CMV infection. UC patients diagnosed with CMV infection had significantly longer disease duration. CMV infection rate was 7.9% (n=3) in patients with newly diagnosed ulcerative colitis by PCR. The usage of immunosuppressive therapy is an important risk factor for CMV infection among patients with UC. In two recent meta-analyses, it was stated that CMV is more common in patients with long-standing UC. It has been reported that the length of the disease duration increases the risk of CMV co-existence. It has been stated that the usage of immunosuppressants and steroids such as azathioprine and anti-TNF alpha increases the risk of exacerbation due to CMV in UC cases. In the exacerbation of UC cases with these risk factors, the importance of excluding CMV co-existence with PCR or IHC in colon biopsy for clinical outcome and treatment regulation was stated [19,20].

In our study, it was observed that the presence of CMV worsened the clinical outcome by causing treatment failure. The prevalence of CMV was found to be higher in patients with steroid-dependent or steroid-resistant UC than in non-dependent or non-resistant patients. In CMV-positive patients with UC, steroid resistance or dependence was found more than in negative cases. In logistic regression analysis, CMV was observed to be a risk factor for increasing steroid resistance and dependence. The incidence of steroid resistance varies between 16% and 30% in patients diagnosed with UC [21-23]. The prevalence of CMV infection was observed to be 67% in steroid-refractory moderately and severely active UC patients by using PCR [24]. Although the mechanism of steroid resistance is not known clearly, it is thought that steroid resistance may occur due to the reactivation of CMV infection. In a recent meta-analysis, it was shown that CMV-positive cases have more steroid resistance than CMV-negative cases [25].

The answer to the question of whether CMV is an active pathogen or an "innocent bystander" in patients with UC is controversial. Recent research has shown that CMV may worsen the course of UC. CMV infection is known to increase the severity of UC exacerbations and the risk of exacerbation-related hospitalization [26,27]. Kim et al. studied a cohort of 72 patients with moderate to severe UC evaluated for CMV reactivation; higher rates of colectomy requirement were observed in the CMV-positive group [28]. Also in this study, authors reported that the higher disease exacerbation rate was determined in the CMV-positive group. As a result, patients with UC exacerbated by CMV had a worse prognosis than patients without CMV, and antiviral therapy with ganciclovir significantly reduced the requirement for colectomy in patients with severe UC. They suggested that the high level of CMV viral load played a role in the prognosis of UC. Therefore, CMV status should be tested in patients using immunosuppressants, especially steroid-refractory or dependent UC [28,29]. In our study, it was observed that the severity of clinical exacerbation was higher in the CMV-positive group. CMV-positive patients with UC had significantly higher CRP and Mayo Score than those without CMV. UC patients diagnosed with CMV infection had significantly more extended disease localization. Despite antiviral therapy, clinical remission and mucosal healing rates were lower in CMV-positive patients than in CMV-negative patients. It was observed that the requirement of colectomy increased due to more severe activation among patients with CMV-positive UC. It has been found that the presence of CMV infection increases the risk of developing steroid dependence or resistance. Based on these results, we can offer that CMV may trigger exacerbations of UC, worsen the outcome of the disease, and increase the requirement for colectomy.

Strengths of our study

It is possible to reach detailed patient data in the periods when the cases were applied actively and in the post-treatment follow-ups and to reach a sufficient number of patients. Although this is a retrospective study, it enabled us to make an adequate and detailed analysis. Risk factors affecting CMV positivity in patients with UC could be determined by analyzing. In addition, the effect of CMV positivity on UC disease severity and treatment outcome, especially on the development of steroid resistance, mucosal healing, and clinical remission could be evaluated.

Limitations of our study

When we evaluate the limitations of our study, the biggest inadequacy is that it is a retrospective study. We believe that in a prospective study, the short- and long-term effects of CMV on UC can be determined more clearly. In addition, since all CMV-positive cases in our study received ganciclovir treatment; we could not clearly see how effective antiviral treatment was on CMV infection. However, despite antiviral therapy, the CMV-positive group had less response to treatment and more obligatory colectomies were observed.

## Conclusions

CMV should be suspected in patients with severe or moderate active UC with treatment resistance, especially in cases with long disease duration and in the presence of extensive colon involvement. The presence of usage of steroids and immunosuppressives such as AZA and anti-TNF-alpha agents increases the risk of CMV developing. The presence of CMV increases the severity of the disease and worsens clinical outcomes. CMV infection leads to the development of steroid resistance or dependence, reducing the success of treatment. The presence of CMV infection increases the requirement for colectomy. It can be recommended to investigate the presence of CMV in case of steroid resistance/dependence and before the initiation of immunomodulatory or anti-TNF-alpha therapy.
